# Study protocol for the Integra Initiative to assess the benefits and costs of integrating sexual and reproductive health and HIV services in Kenya and Swaziland

**DOI:** 10.1186/1471-2458-12-973

**Published:** 2012-11-13

**Authors:** Charlotte E Warren, Susannah H Mayhew, Anna Vassall, James Kelly Kimani, Kathryn Church, Carol Dayo Obure, Natalie Friend du-Preez, Timothy Abuya, Richard Mutemwa, Manuela Colombini, Isolde Birdthistle, Ian Askew, Charlotte Watts

**Affiliations:** 1 Population Council, General Accident Insurance House, Ralph Bunche Road, P.O. Box 17643-00500, Nairobi, Kenya; 2 London School of Hygiene & Tropical Medicine, Department of Global Health and Development, 15-17 Tavistock Place, WC1H 9SH, London, UK; 3 London School of Hygiene & Tropical Medicine, Department of Population Studies, Keppel Street, WC1E 7HT, London, UK

**Keywords:** Sexual and reproductive health, HIV services, Integration, Sub-Saharan Africa

## Abstract

**Background:**

In sub-Saharan Africa (SSA) there are strong arguments for the provision of integrated sexual and reproductive health (SRH) and HIV services. Most HIV transmissions are sexually transmitted or associated with pregnancy, childbirth, and breastfeeding. Many of the behaviours that prevent HIV transmission also prevent sexually transmitted infections and unintended pregnancies. There is potential for integration to increase the coverage of HIV services, as individuals who use SRH services can benefit from HIV services and vice-versa, as well as increase cost-savings. However, there is a dearth of empirical evidence on effective models for integrating HIV/SRH services. The need for robust evidence led a consortium of three organizations – International Planned Parenthood Federation, Population Council and the London School of Hygiene & Tropical Medicine – to design/implement the Integra Initiative. Integra seeks to generate rigorous evidence on the feasibility, effectiveness, cost and impact of different models for delivering integrated HIV/SRH services in high and medium HIV prevalence settings in SSA.

**Methods/design:**

A quasi-experimental study will be conducted in government clinics in Kenya and Swaziland – assigned into intervention/comparison groups. Two models of service delivery are investigated: integrating HIV care/treatment into 1) family planning and 2) postnatal care. A full economic-costing will be used to assess the costs of different components of service provision, and the determinants of variations in unit costs across facilities/service models. Health facility assessments will be conducted at four time-periods to track changes in quality of care and utilization over time. A two-year cohort study of family planning/postnatal clients will assess the effect of integration on individual outcomes, including use of SRH services, HIV status (known/unknown) and pregnancy (planned/unintended). Household surveys within some of the study facilities’ catchment areas will be conducted to profile users/non-users of integrated services and demand/receipt of integrated services, before-and-after the intervention. Qualitative research will be conducted to complement the quantitative component at different time points. Integra takes an embedded ‘programme science’ approach to maximize the uptake of findings into policy/practice.

**Discussion:**

Integra addresses existing evidence gaps in the integration evaluation literature, building on the limited evidence from SSA and the expertise of its research partners.

**Trial registration:**

Current Controlled Trials NCT01694862

## Background

There are many well-established reasons that support the rationale for integrating or linking sexual and reproductive health (SRH) and HIV services in developing countries with generalized HIV epidemics – primarily in sub-Saharan Africa. Most HIV transmissions in these countries are sexually transmitted or associated with pregnancy, childbirth and breastfeeding. Many of the behaviours that prevent HIV transmission also prevent sexually transmitted infections (STIs) and some prevent unintended pregnancies. Individuals who use SRH services often benefit from HIV services, and vice versa, and so by integrating services, there is the potential to increase the coverage of both HIV and SRH services. There are an estimated 34 million people living with HIV worldwide at the end of 2010, up 17% from 2001 [1], and around two thirds of people living with HIV are found in sub-Saharan Africa. Globally, almost 90 million women have an unintended pregnancy each year largely due to an unmet need for family planning (FP) [2]. Findings from several studies in countries south of the Sahara have also suggested that rates of unintended pregnancies among HIV-infected women are high ranging from 51% to 91% [3-5][6]. There is a clear need to respond to the sexual and reproductive health needs of people living with HIV.

Over the past decade, a number of international statements, position papers and advocacy efforts have indicated the commitment of the international community to intensifying linkages between SRH and HIV, at both policy and health service delivery levels [7,8]. In Africa, the Maputo Plan of Action affirmed the need to act [9]. The 2005 interagency document developed by WHO/UNFPA/UNAIDS/IPPF and entitled *“Sexual and Reproductive Health and HIV, A Framework for Priority Linkages”*[10], outlined some of the ‘expected’ benefits, including improved access and efficiency of services and better health outcomes, and recognized the imperative for improving the evidence-base in order to ascertain the magnitude of these benefits. A call echoed at an international conference organized by the Bill and Melinda Gates Foundation (BMGF) in Ethiopia, entitled *“Linking Sexual and Reproductive Health, Family Planning and HIV”*[11].

In addition to these political statements and calls for evidence, four comprehensive reviews of integration literature have surveyed existing evidence. They found a range of potential benefits of integrating reproductive health and HIV services, including improvements in service utilization (such as increase in uptake of HIV testing and contraceptive use), quality of services and client satisfaction, but acknowledged many gaps in the evidence, especially for impact of integration on health outcomes and costs [12-15]. The reviews also highlight a wide range of challenges associated with integration, including provider overload and limited time, poor or insufficient provider training and motivation, and stock-outs of essential drugs and supplies.

There are a limited number of evaluative studies for integrating HIV services with components of SRH services, mostly from southern and eastern Africa where HIV prevalence is highest. Early studies on integration in sub-Saharan Africa highlighted the complexity of ensuring that the service structures, funding and commodity flows adequately supported the provision of integrated services, and found mixed evidence of clear benefits in terms of the new uptake of services or the better use of resources [16,17]. Two more recent studies from South Africa and Kenya suggest more positive results. Using quasi-experimental designs to evaluate models for integrating HIV services (STI/HIV counseling and risk assessment, HIV testing) into services for FP clients, both studies found that integration led to an increased proportion of providers discussing and offering HIV counseling and testing during FP consultations, and increases in the quality of care. Both studies also found that integrating STI/HIV counseling and offering HIV counseling and testing was feasible and acceptable to FP clients and providers [18,19]. Two other studies, in Kenya and Swaziland, evaluated models for linking and strengthening HIV screening and management and postpartum family-planning into services for postnatal clients and their newborns. Both found significant improvements in process outcomes, including uptake and quality of services and confirmed that integrated services are feasible and acceptable to both postpartum women and health care providers [20-22]. However, none of the above studies in Kenya, South Africa or Swaziland specifically focused on measuring the benefits of integrated HIV and SRH services; nor included comparison facilities nor assessed the communities’ response to these services. These studies need to be replicated before any broader conclusions can be drawn on the benefits of integration to service uptake and quality; moreover the studies lacked evidence on impact for health outcomes and service costs.

The Integra Initiative addresses these gaps and builds on this evidence from sub-Saharan Africa, as well as the expertise of its research partners. Since a partial evidence base exists for integration of HIV services (counseling, testing, risk assessment, care) into family planning and postpartum/postnatal services, these services have been selected as the focus of the Integra Initiative’s work which is in Eastern and Southern Africa – the regions with greatest need for HIV services.

### Study aims, hypotheses, objectives and indicators

The overarching aim of the Integra Initiative is to strengthen the evidence base on the impact of integrating family planning (FP), postpartum/postnatal care (PNC) and HIV services in sub-Saharan Africa. Specifically, in the study, we aim to assess the following three objectives:

1. To assess the extent to which different models of integrated service provision increase the range, uptake and quality of selected SRH and HIV services, and lead to a greater diversity in the profile of clients

2. To explore whether the provision of integrated HIV and SRH services leads to reductions in HIV risk-behavior; HIV related stigma and unintended pregnancies

3. To assess the efficiency of using different operational models for delivering integrated services in terms of: cost, utilization of existing infrastructure and human resources

### Definition and types of integration models

**Figure 1:**
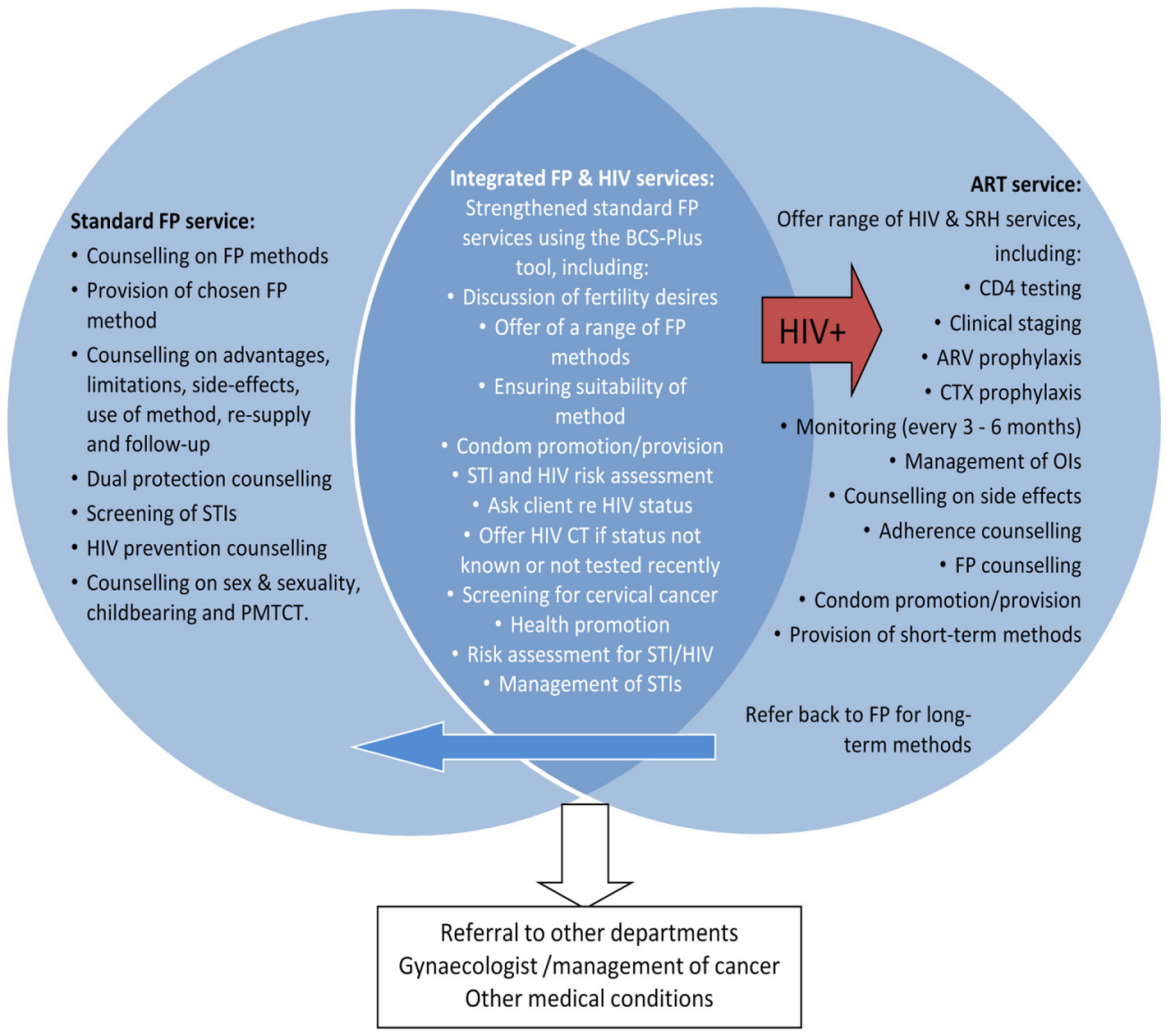
Package of integrated FP/HIV services.

For the purposes of this study integration is defined as offering clients two or more services in the same visit. To better understand how services can be integrated in different countries, this study focuses on two key models of integration in Kenya and Swaziland. The first model focuses on integration of FP and HIV services (integrated FP model) and entails performing HIV testing, STI screening and management, cervical cancer screening, condom promotion within FP consultations, as well as active referral to antiretroviral (ART) units for HIV-positive clients (Figure 1). The FP model will be evaluated in Kenya only.

**Figure 2:**
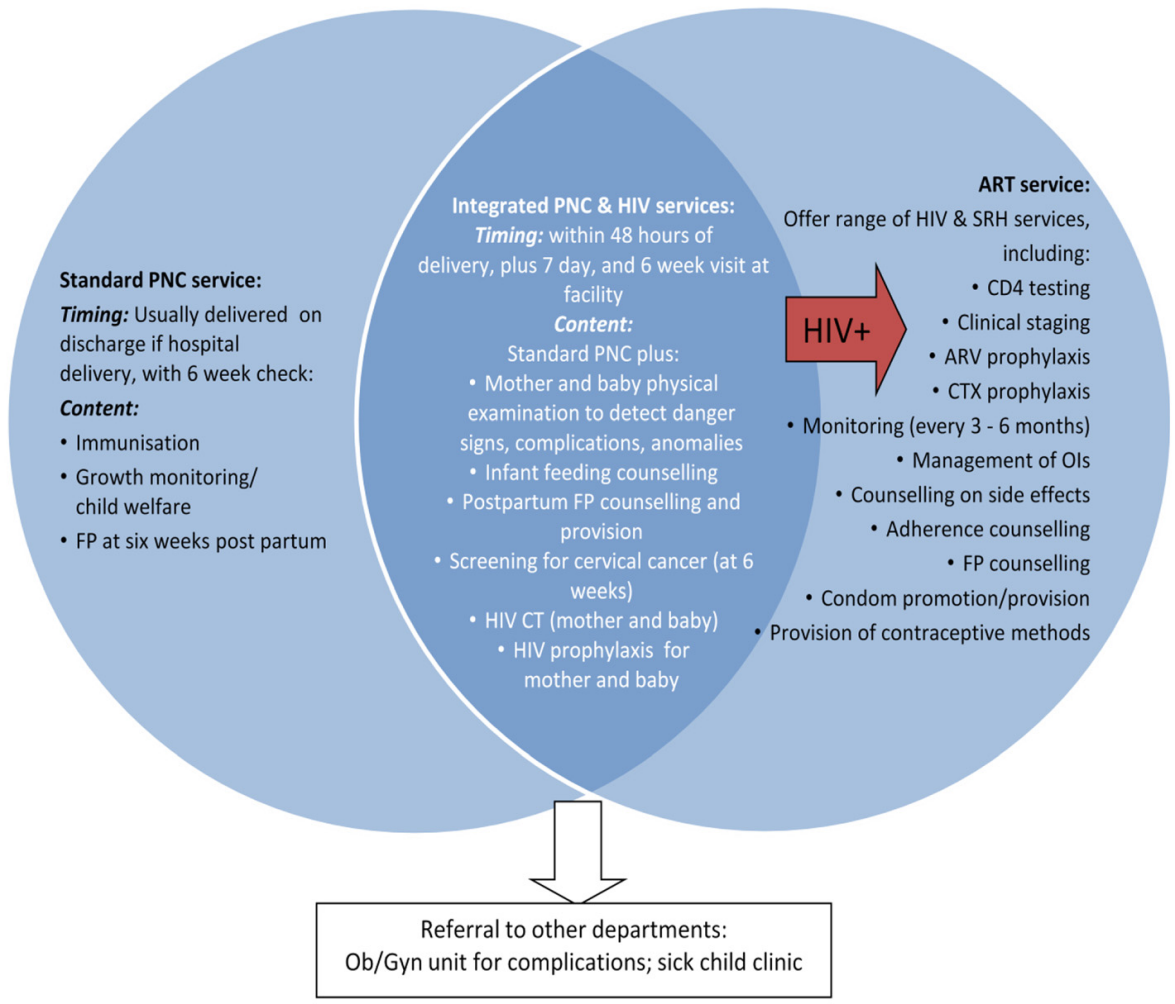
Package of integrated PNC/HIV services.

The second model focuses on integration of PNC and HIV services (integrated PNC model) and will be implemented in both Kenya and Swaziland (Figure 2). The model focuses on the provision of PNC services to mother and baby, FP services, repeat HIV testing for mother, HIV testing for infant and referral to HIV services for HIV positive mothers and infants, as well as referrals for clients requiring additional services.

Two further models will be investigated as sub-studies to the main study which are not described in detail here. The first of these entails integrating FP and HIV/STI services (integrated SRH services model) in International Planned Parenthood Federation-Family Health Options Kenya (IPPF FHOK) and IPPF FLAS (Family Life Association of Swaziland) clinics in Kenya and Swaziland, respectively. This model focuses on the integration of the following services: FP, maternal and child health (MCH), HIV testing, HIV care, STI services, cervical cancer screening, and youth counselling. The second model focuses on the sexual and reproductive health needs of HIV-positive people in Swaziland and compares integrated SRH-HIV with stand-alone HIV services. Findings from these studies will be reported separately and more information on both sub-studies can be found on the Integra Initiative website: http://www.integrainitiative.org

### Description of intervention

Table 1 provides a summary of the programmatic components and related activities that will strengthen and maintain the provision of integrated FP/PNC and HIV services; introduce new services and develop and strengthen the linkages between the services that were not integrated at intervention facilities. Existing standard services will be compared with the strengthened integrated FP and PNC service models shown in Table 1.

**Table 1:** Summary of the interventions in FP and PNC sites

**Intervention component**	**Model 1: FP-HIV Kenya**		**Model 2: PNC-HIV**
Pre-existing standard services	·Counselling & provision of FP methods		·**Timing:** Usually delivered on discharge if hospital delivery, with 6 week check:
	·Dual protection counselling		·**Content:**
	·Screening of STIs		·Immunisation
	·HIV prevention counselling		·Growth monitoring/child welfare
	·Counselling on sex & sexuality, childbearing and PMTCT.		·FP at six weeks postpartum (on request from mother)
	·Refer FP clients for STI treatment/ syndromic management		(NB: this is what should happen according to the guidelines but rarely does at facility level)
	·Refer FP clients for HIV counseling and testing		
	(NB: this is what should happen according to the guidelines but rarely does at facility level)		
Strengthened or additional services introduced	·All clients receive strengthened Standard FP services using the Balanced Counseling Strategy Plus. This includes:		·**Timing:** within 48 hours of delivery, plus 7 day, and 6 week visit at facility
	·Discussion of fertility desires,		·**Content:**
	·Offering a range of FP methods		·Mother and baby physical examination to detect danger signs, complications, anomalies
	·Ensuring suitability of FP methods		·Infant feeding counseling
	·Condom promotion/provision		·Postpartum FP counseling and provision
	·STI and HIV risk assessment		·Screening for cervical cancer (at 6 weeks)
	·Check client HIV status		·HIV counselling & testing (mother and baby)
	·HIV counseling and testing		·HIV treatment prophylaxis for
	·Screening for cancer of cervix (via /vili)		·mother and baby
	STI screening and management Arrange follow up appointments		Neonatal male circumcision (Swaziland only)
	**HIV negative clients** Health promotion Risk factor exposure Risk assessment (routine) for STI/HIV		
	**HIV positive clients** Counseling on HIV care and treatment available Manages clients with CD4 count >350 CTX prophylaxis Referred to lab/ART unit for blood tests		
Adaptation/ strengthening of protocols, guidelines and training materials	·Development, adaptation or updating of guidelines and protocols (where necessary) based on national guidelines and manuals		
	·Mentoring toolkit developed - includes trainers guide, log book for mentees, checklist		
	·Balanced Counseling Strategy Plus second edition adapted to include cervical cancer screening and postpartum car		
Staff training & management	·Development of an appropriate training and monitoring/ supervisory package which was grounded on the mentorship methodology and used the Balanced Counselling Strategy Plus (BCS+) toolkit		
	·Facilitative supervision comprising initially of bi-monthly and later quarterly visits by supervisory teams from the Ministries of Health and Population Council		
	·Training on technical skills for provision of long acting FP methods (IUDs and implants)		
	·Training on HIV counseling and testing, conseling on HIV services available, ARV refills, screening for STIs and syndromic management of STIs		
Organizational change and role clarification	·Organizational change of how services are provided (rooms identified for integrated services, partitions built to create more rooms and staff reallocated		
	·Role clarification with all staff - task oriented work changed to provision of services according to client need		
Equipment & supplies	·Ensuring availability of minimum levels of equipment and supplies (e.g., implant/IUD insertion kits, blood pressure machines and stethoscopes) required for providing integrated services		
Communication aids	·Improving the availability of information, education and communication/behavioral counseling and communication (IEC/BCC) materials. All IEC materials pertaining to FP/HIV and PNC/HIV reviewed and adapted to reflect provision of integrated services		
Referral systems	·Strengthened referral system between SRH clinics and ART centres (introduction of new referral forms (Kenya only)		
Data collection and management	·Strengthening data collection and recording systems through the development of data capture tools, (e.g., PNC registers and monthly data monitoring forms)		

In order to build consensus on the timing and composition of the package of services for both integration models, meetings were held with relevant stakeholders and the different levels of the Ministries of Health (MOH) in Kenya and Swaziland. Nine areas of intervention were identified (see list below) and to be carried out between August 2009 - December 2010 in both countries and for both models.

Summary of nine areas of intervention

1. Adaptation/strengthening of protocols, guidelines and training materials for HIV/SRH (where necessary)

2. Develop an appropriate training, mentoring and supervisory package for on job training

3. Improving provider capacity through mentorship (including technical skills for providing postpartum care for the mother and postnatal care for the infant, long term FP, HIV counseling and testing, HIV services, screening/management for STIs and cervical cancer)

4. Ensuring availability of minimum levels of equipment and supplies required for providing integrated services

5. Support supervision for integrated HIV/FP and HIV/PNC services

6. Organizational change to provide integrated services and role clarification for staff working at intervention sites

7. Improving the availability of IEC/BCC materials

8. Strengthened referral system between MCH-FP clinic and HIV units

9. Strengthening data collection and recording systems.

Guidelines and protocols on HIV, FP and PNC will be reviewed with MOH and stakeholders prior to developing or adapting an integrated package of care as well as the training approach. The training package will be grounded on the mentoring methodology, which is a creative and innovative strategy that prompts the sharing of information and skills among health workers (between a more experienced and less experienced health worker) in order to improve quality of care and/or introduce changes in service delivery. The mentoring approach aims to minimize disruption to ongoing service provision, i.e. one mentor based in a facility will impart her knowledge and skills to her colleagues. The training of mentors will be conducted over a 5-day period to build knowledge and skills in FP and /or PNC and HIV integration. Training job aids will also be reviewed and adapted including the Balanced Counselling Strategy Plus (BCS+) toolkit, a practical, interactive, and client-centered counselling approach that utilises an algorithm, counselling cards and brochures to facilitate FP consultations [23]. Following the introduction of the mentoring approach, supportive supervision will be conducted by the Integra Initiative and MOH officials to promote discussion and implementation of the activities including role clarification, organizational change, strengthened referral/linkages between SRH and HIV services and correct management of service statistics.

While all intervention activities will be uniformly delivered across the study sites, where necessary the activities will be customised to fit the aims and purpose of the two different models under evaluation. To ensure compliance or adherence with the intervention activities, supervision visits and facility meetings (with staff and management committees) will be conducted by teams comprising representatives from relevant departments at the MOHs in both countries and staff members from the Population Council.

### Study setting

The Integra Initiative selected Kenya, a medium-prevalence HIV country, and Swaziland, a high prevalence HIV country as contrasting settings where a partial evidence base existed to build on, and where Integra Initiative partners already had experience in working. The FP model of integration will be implemented in Central Province in Kenya, where facilities serve a population with a high modern method contraceptive prevalence rate (58%) compared with the national average (33%) [24], and slightly lower HIV prevalence (7.6%) among women aged 15 to 49 years in Central Province compared to the national level (8.7%) [25]. The PNC model will take place in Eastern Province in Kenya where facilities serve a population with a relatively high modern method contraceptive prevalence rate (48%), and relatively low HIV prevalence (5%) among women aged 15 to 49 years. The national HIV prevalence among pregnant women is 9.7% [25]. In Swaziland the PNC model of integration will take place in facilities that serve a population with a relatively high modern method contraceptive prevalence rate (48%), compared to 36% nationally; a similar HIV prevalence among women aged 15 to 49 years (31%) compared to national average of 26%, and 39% among pregnant women compared to the national rate of 38% [26].

### Methods/Design

#### Approach and conceptual framework

**Figure 3:**
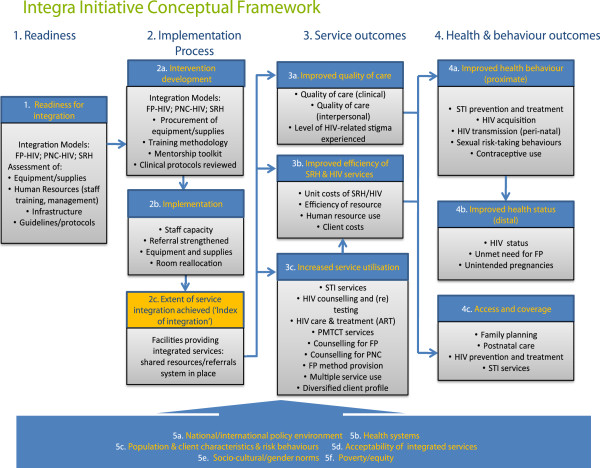
Integra Initiative Conceptual Framework.

The implementation environment for the Integra Initiative is complex, with government policies and practices around integration in constant development. The Integra Initiative therefore embeds rigorous evaluation research within the ‘reality’ of government commitment to large-scale delivery of integrated HIV and SRH services, and to use the findings to strengthen ongoing service provision at each site. The Integra Initiative has developed a conceptual framework (Figure 3) to guide its evaluation, based on a logic model encompassing inputs, processes and outcomes, as well as the wider community and policy context. This model encompasses different components and processes of integration, ranging from readiness and service infrastructure, delivery, utilization and outcomes along with the needs and perspectives of providers, clients and community members.

There is an emerging body of literature addressing the challenges of using randomized controlled trials to assess the impact of public health interventions [27-29]. Particularly in cases such as the Integra Initiative, where the causal chain between intervention and outcome is long, where there is a broad range of outcomes that need to be explored, and where there is already a degree of integration occurring in some clinic settings, attempting to conduct a randomized controlled trial is not appropriate [27-29]. Consistent with evaluation designs described by Habicht and colleagues [27], the Integra design includes evaluation of performance and impact to try to make two types of causal inference: adequacy and plausibility. Evaluation of adequacy will assess whether the expected changes in provision, service utilisation and cost-effectiveness have occurred in intervention facilities (Columns 1 & 2 of the logic model). Evaluation of impact will assess the plausibility that changes in service, health and behavioral outcomes (Columns 3 & 4) are due to the Integra Initiative. The case for such plausibility will be built from the following strands of evidence:

• Comparing findings in ‘intervention’ facilities with those in facilities chosen as ‘comparison’ sites prior to the evaluation

• Exploring a dose–response relationship between the measured extent of integration and the study outcomes

• Measuring changes in performance over time, to demonstrate a logical sequence between the intervention (integration) and outcomes

• Measuring change in each step of the logic model – a prerequisite for any attribution to the intervention

• Triangulating findings from a mix of research methods to capture a range of perspectives and insights from different disciplines

The study will employ a controlled pre- and post-test quasi-experimental, or non-randomised, design. Since the research is being conducted in real-life health delivery settings where programmatic contamination is possible due to ongoing health programme interventions over the study period, the control group will be referred to as a ‘comparison group’, for which outcomes will be compared over time up to two years after implementation.

#### Selection of intervention study sites

The study will be implemented in public health facilities in Central and Eastern provinces in Kenya and in three regions in Swaziland. Six intervention facilities from two districts in Central Province were selected from the original 23 facilities participating in the previous integration study using FP as the entry point [30] and building on that experience. The two criteria for selecting intervention facility selection were: i) good performance in the previous study and ii) high throughput of FP clients (≥100/month). These six intervention facilities include two hospitals and four health centres. In order to match these sites with six comparable sites in the same province and avoid contamination, additional criteria (see below) were used to randomly select facilities with similar characteristics from outside the two original study districts.

The existing four facilities (public health units/MCH-FP) from the previous study in Swaziland [31] were selected as the intervention facilities. In order to match these sites with four comparable sites and avoid contamination, criteria were used to select the comparison facilities that have similar characteristics but away from original study sites. Sites that fitted the criteria (see below) were visited in July 2008 by Population Council, London School of Hygiene & Tropical Medicine (LSHTM) and the MOH and assessed for comparability to the intervention sites. In discussion with the Provincial Health Management Team for Eastern Province, Kenya we were advised not to continue with the district from the previous study [20]. This was due to the high number of other health sector interventions. Therefore, two districts with limited external support were selected and randomly allocated as intervention and comparison sites.

#### Selection of comparison study sites

To maximize equivalence between the intervention and comparison facilities, thereby minimizing selection bias, a pair-wise matching sampling design will be used. Comparison facility selection will be based on four criteria, namely: high client load (> 50 infants/month receiving their first immunizations at 6 weeks at the PNC-HIV clinics and >100 FP clients/month at the FP-HIV clinics); a minimum of two providers qualified in and currently delivering FP services; a range of services available (FP, voluntary counseling and testing (VCT), STI treatment, prevention of mother-to-child transmission (PMTCT); and no current provision of fully integrated PNC-HIV and FP-HIV services. Facilities allocated to each study model serve populations with similar socio-economic characteristics, have a similar health infrastructure (i.e. public sector), and serve a population with a relatively high modern method contraceptive prevalence rate and similar HIV prevalence among women aged 15 to 49 years. To minimize contamination, the facilities’ (with comparable characteristics) will be selected in different districts in the same province. The number of facilities that will be purposively selected for each model and country are summarized in Table 2.

**Table 2:** Sampling strategy and sample size calculations for each Integra research component

**Research Component**	**Sampling strategy**	**Key indicators used in sample size calculation**	**Adjustments to sample calculation**	**Model 1. Kenya FP**	**Model 2. Kenya PNC**	**Model 2. Swaziland PNC**
Sampling of facilities	Theoretical sampling to point of saturation within province	4 criteria: high client load; minimum of 2 FP providers; range of SRH services available; no current provision of SRH-HIV integration	n/a	12 facilities: 6 Intervention and 6 Comparison	12 facilities: 6 Intervention and 6 Comparison	8 facilities: 4 Intervention and 4 Comparison*
HFAs: Facility inventory				1 per facility	1 per facility	1 per facility
HFAs: Client exit interviews & linked observations of consultations	Consecutive sample and saturation	Quality of Care analysis 6 CPIs	No	18 per facility: 6 new clients, 6 repeat clients and 6 clients switching FP method	6 within 48 hours postpartum 6 at one week and 6 at 6 weeks per facility	6 within 48 hours postpartum 6 at one week and 6 at 6 weeks
HFAs: Structured interviews with providers	Convenience	All providers working in MCH-FP units	No	3-6 providers per facility	3-6 providers per facility	3-6 providers per facility
HFAs: Client flow assessment	Census over 5 days based on existing client load		No	50 – 200 clients per day	50 – 200 clients per day	50 – 200 clients per day
Cohort	Consecutive sample of female clients (recruited if within 12 weeks post-partum in PNC facilities)	Kenya: 80% power to detect a 5% increase in condom use, among all women in PNC and those using other contraceptive methods in FP facilities.	1952 clients: 976 from Intervention and 976 from Comparison facilities			
	Swaziland: To detect a 7% increase in condom use among PNC clients.	30% loss to follow up; over-sampling of HIV-positive clients until min of 400 in Intervention & Comparison facilities	1978 clients: 989 from Intervention and 989 from Comparison facilities			
Household survey	3-stage cluster survey design, for random selection of EAs, households and individuals	80% power to detect 10% increase in % of women who have ever used study facility.	Design effect of 2.0	1632 (816 male; 816 female) within 10km catchment area of FP facilities	n/a	816 (408 male; 408 female) within 10km catchment area of PNC facilities
***Qualitative – in-depth interviews***						
Providers	Convenience sampling			1-3 providers per facility	1-3 providers per facility	1-3 providers per facility
Cohort clients	Purposive sampling to reflect issues emerging from quantitative data		25 HIV-positive clients; and 25 other clients	25 HIV-positive clients; and 25 other clients	25 HIV-positive clients; and 25 other clients	
Community members	Purposive sampling to reflect issues emerging from quantitative data		20 men	n/a	20 men	

* 2 facilities subsequently added due to lower than expected client load at comparison sites.

#### Overview of study components

In order to address the objectives, the study will include three levels of analysis. The first unit of analysis will be the facility level. Facility-based research will be conducted at four stages during the study in order to track changes over time in clinic preparedness for the range of services offered, service integration and utilization, quality of care indicators, client satisfaction, costs and efficiencies of service provision. Health Facility Assessments (HFAs) will collect comparable data across clinics, incorporating the following methods: facility checklists, structured observations of client provider interactions (CPI), client exit interviews, health care provider interviews, client flow analysis, routine program and costing data analysis. The second unit of analysis will be the client level and will involve research to understand how the demand for and use of SRH and HIV services is shaped by the changing structure and performance of facilities, and how integration impacts upon client SRH and HIV-related behaviours. This will be done through a variety of methods, including: client exit interviews and a cohort study of clients to assess how individual outcomes are shaped by the services accessed (including fertility intentions, unintended pregnancy, use of FP, HIV status, STI/HIV-related behaviors and health seeking behaviors). In depth interviews will also be conducted with a sub-sample of cohort clients to explore experiences and motivations for use of services, facility switching, stigma and other issues arising from initial quantitative analysis. The third unit of analysis is the population level, which will involve household surveys within communities living in the catchment areas of selected study facilities. This will allow comparison of the characteristics, behaviour and attitudes of clients *vs.* non-clients, as well as offering insights into demand for and use of for vertical or integrated services and reasons for use/non-use of these. In-depth interviews (IDIs) and focus group discussions (FGDs) with groups of men comprising service users and non-users will also be conducted. These qualitative interviews with community members can allow more detailed exploration of reasons for service-use patterns, choice of facilities and reasons for non-use.

#### Sampling and indicators

Details on research methods are provided below; sampling strategy and sample size calculations for each method are summarised in Table 2.

The operational results (outcomes) and the main indicators that will be measured and analysed for each study objective are shown in Table 3.

**Table 3:** Operational results and indicators to be used to compare results from the intervention and comparison health facilities

**Objective 1. Determine the benefits of different integrated models to increase range, uptake and quality of selected SRH and HIV services and to diversify the profile of clients.**		
**Results/Outcomes**	**Indicators**	**Data source**
Feasibility of linking services demonstrated	Number of internal and external referrals, Number of staff updated in HIV counseling and testing Number of staff updated in HIV BCC prevention, Facilities have adequate equipment and supplies	Service statistics Provider interviews Facility inventory
Provision of linked services to clients	Clients received CT in FP consultation, Clients referred for ART, Clients referred for other services, Number of referrals attended, Proportion of population demanding integrated services, Proportion of population accessing integrated services, Proportion of services users giving integration as a reason for choice of clinic	Client exit Provider interview Cohort Community Survey
Increased % of eligible HIV+ women starting to use ART	Proportion of women reaching referral site clients know their CD4 counts	Service statistics Cohort
Increased uptake of range of SRH services including integrated CT, FP, PNC and STI screening services	% FP clients accessing more than one service Proportion of population accessing more than one service at last FP or PNC visit	Service statistics Exit /Cohort Community Survey
Increased quality of a range of SRH services	% clients receiving minimum level of quality services	Client – provider interaction, exit interview
Increase in number and diversity of clients	% men and women using component services Proportion of clients using component services by age, SES and gender	Community Service Statistics Client exit /CPI
Increased numbers of clients screened/managed for STIs	% FP clients accessing separate/integrated services	Service statistics Client Flow Cohort
Increased in new and repeat FP clients testing for HIV		Service statistics Cohort
Improved attitudes of service providers towards HIV+ clients	% Providers indicating non discriminatory attitudes % Clients recommending services to others	Provider interview Cohort survey Community Survey
**Objective 2. Determine the impact of different integrated services on changes in HIV risk-behavior; HIV related stigma and unintended pregnancies.**		
Reduction in reported HIV risk behaviors among HIV negative & HIV positive clients	Condom use at last sex; Number of partners in past 12 months Received STI/HIV counseling Consistent condom use reported Use of condoms with another FP method (dual protection)	Cohort Client Exit Community Survey
Reduced incidence of unintended pregnancies	% women who become pregnant (incidence) % women reporting planned pregnancy % women with correct knowledge of fertile period % of population who report unintended pregnancy in last 12 months	Cohort Client Exit Community Survey
Increased duration of contraceptive use among HIV negative & HIV positive clients	Ever/current use of FP method Discontinuation FP rates in 12 months Ability to achieve fertility goals	Cohort survey
Improved attitudes of service providers towards HIV+ clients	Proportion of providers indicating non discriminatory attitudes towards HIV positive clients	Provider interview
Reduced stigma at health facilities.	Clients reporting positive experience of CT process % clients reporting unacceptable stigmatizing behavior by providers	Community and Cohort survey
Decreased stigmatization at community level of HIV services if integrated with FP RH services	Perceived barriers to accessing services: costs, distance, quality, waiting times, stigma surrounding service	Community survey Cohort survey
**Objective 3: Establish the efficiency of using different operational models for delivering integrated services in terms of: cost, utilization of existing infrastructure and human resources.**		
Total resource requirements and unit costs of intervention	Cost per eligible client receiving ART, Cost per person-month of receiving ART Cost per client counseled, tested and receiving results Cost per client receiving each service component	Economic study

### Description of methods and data collection

a) Health facility assessments

Four HFAs [32] will be conducted over one month at four time periods between 2009 and 2012 to assess quality of care in study facilities. An initial pre-intervention assessment will be undertaken in both intervention and comparison health facilities to determine the comparability of the facilities. An immediate post-intervention assessment will be undertaken six months post-intervention to evaluate changes in quality of care. The third and fourth HFAs will be undertaken at 18 months and 30 months post-intervention, respectively. HFA components comprise a facility inventory, review of service statistics, interviews with health care providers, observations of client-provider interactions (CPIs), client exit interviews and client flow data. Data collection procedures for each component of these assessments are as follows:

i) Facility Inventory

An inventory will be taken of available resources required to deliver the intervention, including infrastructure, staffing numbers and skills mix, services provided, staff training undertaken, availability of equipment, commodities, forms and registers (client cards and notes), medications Researchers will request the head of the facility to guide them around to observe and record relevant information on a checklist.

ii) Interviews with healthcare providers

Structured interviews with health care providers will be conducted to determine their knowledge and skills for FP, PNC, HIV counseling and testing and other STI/HIV services; their understanding of organizational structure and related activities; their perceptions of barriers and operational challenges to FP/PNC clients’ acceptance of HIV counseling and testing; and their attitudes towards policy and procedural changes needed for provision of integrated care. All providers in the MCH-FP and ART units will be approached for interview in the 24 study facilities in Kenya, and 8 facilities in Swaziland. We anticipate between 3 and 6 providers from each facility will be interviewed.

iii) Observations of client-provider interactions

This component entails a structured non-participatory observation of health consultations, encompassing both the counseling process (how clients are treated and whether they actively participate) and the technical content of a consultation (provision of essential information and its technical accuracy). To reach a meaningful measure of quality of care with a relatively small sample size for the FP-HIV model, 18 consecutively sampled new FP clients and 18 revisit FP clients (assuming 6 clients for an average of three health providers in each facility) will be observed. For the PNC-HIV model in Kenya and Swaziland, 24 consecutively sampled postpartum women (between 6 to 8 postpartum women observed: within 48 hours of birth, between one to two weeks and around six weeks postpartum) per study facility. We acknowledge that observing client provider interaction may bias in a positive direction the results obtained on quality of care. We will spend more than one day at each site, so that the presence of the research team becomes more familiar and the behavior of the providers becomes more normative, furthermore over the three to four year data collection period the presence of researchers will become very familiar to the staff.

iv) Client exit interviews

Exit interviews will be held with each client who was observed to ascertain their perceptions of and satisfaction with the service received, as well as their SRH and HIV-related behaviors and fertility intentions.

v) Client flow assessment

A five-day assessment of service utilisation patterns will be conducted at each facility, biannually in Kenya, and annually in Swaziland, to measure: the total number of clients at each facility (per day); the service type being sought; the extent of service integration and referrals; and waiting times. Client data will also be captured including age, sex, and residence (proximity to the health facilities). All clients entering the facility for MCH services over the five-day period will be asked to participate, and given a client flow instrument by research assistants and/or service providers. Clients will carry the form throughout their consultations: every service provider seen will complete the form, indicating session start/end times, service(s) received and services referred for.

b) Prospective routine monitoring

This will involve collection of routine program monitoring data on utilization of family planning, postpartum/postnatal and HIV services. Forms will be used to extract client register data on a monthly basis for further analysis. Analysis of routine program data will contribute to answering the following questions: 1) what is the change in client profile pre- and post-integration (for instance, in terms of age, and sex)?, 2) does the rate of new clients accessing the services increase post-integration?, and 3) what are the changes in range of service uptake and client-profile pre- and post-integration across a range of SRH/HIV services.

c) Costing of service delivery across the different models

In each of the study facilities, the costs of delivering integrated SRH-HIV services will be measured both at baseline and endline, and the potential for efficiency gains will be assessed. Where feasible these cost data will be combined with an effectiveness analysis (with client and population level data) to estimate the cost-effectiveness of integrated services.

A periodic activity review (PAR) tool will be used to provide a mapping of how resources are combined to produce integrated HIV and SRH services in each study facility at baseline. The tool will include questions on facility characteristics, staffing types and levels, scope and number of services offered, clinical practice, descriptions of client flow, and overall description of how integration of services works from the provider’s perspective. Building on the PAR, the unit cost study will estimate the quantity and value of resources that are used to implement activities at facility level. Costing tools will be developed from the first round of data from the PAR, but will generally be based on UNAIDS costing guidelines: *The Costing Guidelines for HIV Prevention Strategies*[33].

A standard ingredients approach will be used. Bottom up methods will be used to estimate the direct costs of each service, and these will be combined with top-down method for the costs of overhead resources. In hospital settings, a step-down approach will be used for costs estimated through the top-down approach. The findings will be used to explore whether there are economies of scope and scale associated with the provision of an integrated package of SRH services, and to explore the relative efficiency of different models of provision. The cost data will be used to conduct an econometric analysis to estimate the impact of different cost drivers, including the extent of integration. This is the first time an economic study of this type will be used to evaluate integrated services [34].

d) Cohort study of clients

After the completion of the post-intervention HFA, two samples of clients accessing PNC and FP services (from intervention and comparison facilities) will be recruited and followed over a two-year period, interviewed up to 4 times. Various indicators will be monitored in these populations, including: condom and contraceptive use, fertility intentions, use of PNC and FP services, HIV status, unintended pregnancy, and SRH behaviors. To be eligible for inclusion in the FP-HIV study, the women will be aged 15 years and over, be revisit FP clients, be living in the catchment area of the health facility, and willing to give their informed consent to be interviewed. For the PNC-HIV model, the women will be aged 15 years and over, be clients attending a postnatal check for themselves and/or their infant (0–10 weeks), be living in the catchment area of the health facility, and willing to give their informed consent to be interviewed.

Cohort recruitment: Table 2 summarises the sample sizes that will be sought for the cohort studies. The minimum sample size will be based on 80% power to detect an increase in reported condom use (the key indicator/outcome that required the largest sample) from 5% to 10% in Kenya, with estimates of treatment effect derived from other studies in Kenya [18,20]; and from 13% to 20% in Swaziland, based on estimates from the 2006–2007 Swaziland Demographic and Health Survey (SDHS) [26]. The other outcome to be measured from these data is unintended pregnancies; since this required a smaller sample size it is covered within the condom use sample.

Cohort follow-up: Follow up interviews will be conducted at 6, 18 and 24 months after recruitment, either in the facility or at the woman’s home. If the woman is not located after three attempts, she will be considered lost to follow-up.

e) Household surveys

Community-based household surveys will be conducted with a randomly selected sample of men and women aged 15–49 years from the catchment communities surrounding the study facilities in Central Province (Kenya) and Manzini town in Swaziland. In Kenya, Thika district (intervention sites) and Nyahururu district (comparison sites) will be surveyed; in Swaziland, only Manzini (the largest urban settlement) will be surveyed where clinics included both intervention and comparison sites. The objective is to gain a representative view of service use around both intervention and comparison facilities and the population level need and demand for services. Use of PNC, FP or HIV/ STI-related services will be measured, as well as the use of informal or traditional health services. Community members will be asked standardized, and sex-specific, questions on access and use of services, attitudes to services, stigma, reasons for service use/non-use, as well as on unmet need for HIV testing, PNC and postpartum FP.

A three-stage cluster survey design will be employed. The primary sampling units (PSUs) will be census enumeration area (EAs), and the sampling frames will comprise the catchment areas of the relevant facilities (≤10km radius). Firstly, probability proportional to size (PPS) sampling will be used to randomly select EAs, with sampling frames derived from national censuses. It is estimated that between 25 and 40 EAs will be required to achieve the sample size in each survey district. Secondly, a random sample of households will be selected within each EA, using a systematic approach to achieve full geographic coverage of the EA based on estimated household numbers. Thirdly, one person will be randomly selected using a household roster to determine the sampling frame of eligible household members. The sample size calculations are displayed in Table 3, and were determined by measurement of the following key outcomes: (i) % respondents who have used a study facility (ii) % who can list the SRH/HIV services available at study facilities, (iii) % of female respondents using multiple services vs an integrated service, and (iv) % of female respondent’s not using modern contraception. The sample calculations were based on 80% power and a design effect (DEFF) of 2.0 to account for clustering, as well as accounting for non-response.

f) Qualitative study

In-depth interviews (IDIs) will be conducted to explore key study themes in greater depth. Respondents will be a sub-sample of cohort clients; health providers in study facilities; and a sub-sample of respondents from household surveys. The purpose of the in-depth interviews with the cohort clients is to explore experiences and motivations for use of services, facility switching, stigma and any other issues that arise after initial quantitative analysis. The purpose of the interviews with healthcare providers is to identify clinic- and provider-specific issues that may affect the delivery of integrated care. Interviews will aim to establish providers’ expectations regarding integration, to develop an in-depth understanding of their ongoing experience with providing integrated SRH-HIV services, attitudes towards PLWH, and to explore specific issues of workload, supervision and support, emotional strains, satisfaction and clinical records.

Interviews with community members are focused on men, since the facility-based client data are predominantly from women. Interviews aim to gain a deeper understanding of various issues, including reasons for service-use patterns, choice and perceptions of different facilities, reasons for non-use (including service-related stigma), and contraceptive and sexual health behaviours, including communication with partners and other community members about care-seeking decisions. The breakdown of the expected number of IDIs by different demographic groups is shown in Table 2.

### Data management and analysis

Statistical analyses will be both cross-sectional and longitudinal, according to the specific study component under analysis. Descriptive analyses will explore distributions and baseline associations between key exposure (provision or use of integrated services), outcomes and potentially confounding variables. Multivariable logistic regression modeling will be used to analyse study outcomes in HFA data (CPI observation data and client exit interviews), client cohort data, client flow data, community survey data and periodic activity review data.

To measure the magnitude of changes in the quality of services provided, composite summary scores will be developed from a series of key items assessed during CPIs. The composite score will be developed from a set of activities providers are required to perform or ask during history-taking for each CPI observed. The items will be based on an accepted standard of quality adapted from the national guidelines. Analyses of facility data will be undertaken and the average mean scores and the proportion of women receiving an acceptable quality of service will be calculated from the composite scores for each set of indicators.

Survival analysis will be conducted with the client cohort data to evaluate the effect of integration on key outcomes over time (up to 24 months follow-up), using two approaches: 1) comparing the cumulative probability of outcomes among clients who received services in intervention versus comparison clinics at the time of recruitment (i.e. an intent-to-treat analysis); and 2) assessing a dose–response relationship between clients’ cumulative exposure to integrated services over the duration of the study.

To evaluate exposure to integrated services, an ‘index of integration’ will be developed. A generalized latent variable will be modeled using data from the client flow analysis and costing tools and validated by testing it against expert opinion (both researchers and service practitioners), to give each clinic a ranking score for its level of integration at various points in the study cycle. The index will be used to: a) assess changes in the extent of integration within each facility over time (whether originally allocated as intervention or not); b) control for baseline differences in integration when comparing outcomes in intervention and comparison facilities; and c) measure changes in the extent of integration over time and apply as a time-varying exposure in the dose–response analysis described above.

Qualitative data will be captured in digitally recorded files, transcribed and translated (where needed), before being exported into QSR Nvivo 8 software for management and analysis. A thematic framework will be used in qualitative analysis, allowing a fusion of both a deductive approach, with themes pre-identified from the study objectives, and an inductive approach, with themes emerging from the data incorporated into ongoing analyses.

The economics findings will be used to explore whether there are economies of scope and scale associated with the provision of an integrated package of SRH-HIV services, and to explore the relative efficiency of different models of provision. Cost data will be captured in specially designed Excel spreadsheets. These will then be entered into a STATA dataset. The impact of integration on efficiency will be assessed using a combination of parametric and non-parametric methods. Descriptive cost and resource use analysis, uni-variate analysis and Data Envelopment Analysis (DEA) will be used to identify those clinics that are more or less efficient; changes in unit costs, resource use, resource sharing and efficiency between baseline and endline; and to explore possible explanations for efficiency changes. Intervention and comparison sites will be compared, but also within these different sites with one another.

In addition, a multi-level multivariable logistic regression analysis will be used to explore any association between unit costs, the intervention and integration. This will first explore the relationship between patient level characteristics and costs (derived from a combination of cohort data with cost data) and then, taking this into account, between facility level characteristics and costs. The integration index will be included as a facility level characteristic. Data from the cohort study will provide client level characteristics and service use. This will be combined with the unit cost data to estimate client level (health service) costs. Data from the periodic activity review data and health facility assessments will be used to define facility characteristics.

### Ethical issues

For all tools researchers will be trained on conduct of ethical procedures and will be monitored during fieldwork by Population Council staff in the field.

Informed consent will be obtained separately for each study participant for each component. Where adolescents aged 15 to 17 years are sampled, they will only be interviewed once parental consent has been obtained. All participants will be given detailed information about the study including: aims/methods of study; institutional affiliations of the research; anticipated benefits, risks/discomfort it may cause (expected to be minimal) and follow-up of the study; the time the questionnaire or interview will take; the fact that they may choose not to answer any questions and that they have the right to abstain from participating in the study, or to withdraw from it at any time, without reprisal; measures that will be taken to ensure confidentiality and anonymity of information provided; the conduct of interviews in places of the participant’s choosing and which maximize audio privacy; contact details of the study coordinator for any questions or concerns.

All data will be stored in password protected computer files. Hard copies of questionnaires, anonymised transcriptions and tapes of the group discussions will be stored securely in a locked cabinet, in accordance with the Population Council policy and the Kenya and Swaziland Data Protection Policies.

### Ethical clearance

The research protocol has been reviewed by key stakeholders and ethical clearance has been granted by the Kenya Medical Research Institute (KEMRI) Ethical Review Board (approval number 113 (integrated FP model) and 114 integrated PNC model), the Scientific Ethics Committee of the Swaziland Ministry of Health (MOH) (approval number MH/599C), the Ethics Review Committee of the London School of Hygiene & Tropical Medicine (LSHTM) (approval number 5426) and the Population Council institutional review board (IRB – approval number 443 (integrated FP model) and 444 (integrated PNC model). The Integra Initiative is registered on the Clinical Trials registration site: ClinicalTrials.gov Identifier: NCT01694862.

## Discussion

Linking programmes for SRH and HIV/AIDS should never be pursued simply for the sake of integrating services, but the evidence base for the public health impact of integrating services, compared with continuing to provide them separately, is still limited. The data generated through this study will demonstrate the extent to which reorganizing models of HIV and SRH services can directly influence some of the key MDG indicators, against which countries, development partners and global institutions are measuring their progress towards meeting the MDGs. The Integra Initiative seeks to address the existing evidence gaps in the integration evaluation literature, building on the limited evidence from sub-Saharan Africa and the expertise of its research partners. Since a partial evidence base exists for integration of HIV services (counseling, testing, risk assessment, care) into family planning and postpartum/postnatal services, these services have been selected as the focus of the Integra Initiative’s work which is in Eastern and Southern Africa – the regions with greatest need for HIV services.

## Competing interests

The authors declare that there are no competing interests.

## Authors’ contribution

CEW is co-PI and was involved in the overall conceptual design and implementation of the project, and overall revision of the manuscript. SM is the lead PI for the Integra Initiative and was involved in the overall conceptual design and implementation of the project, and writing substantial components of this manuscript and overall revision of the manuscript. AV is co-PI and was involved in the design and implementation of the economics and other components of the project, writing components of the manuscript and overall revision of the manuscript. JKK was involved in drafting, re-organizing and overall revision of the manuscript. KC was involved in the conceptual design of the study, writing substantial components and overall revision of the manuscript and in the implementation of the project. CDO was involved in design and implementation of the economics components; NFP was involved in design of the household survey component of the project, writing components of the manuscript and in the implementation of the project. TA contributed to components of the study design and was involved in reviewing the manuscript and the implementation of the project. RM contributed to components of the study design and was involved in reviewing the manuscript and the implementation of the project. MC contributed to qualitative components of the study design and was involved in reviewing the manuscript and the implementation of the project. IB was involved in development of the analysis plan and in writing substantial components of this manuscript and overall revision of the manuscript and implementation of components of the project. IA was involved in the overall concept of the design and implementation of the project. CW is co-PI and was involved in the overall conceptual design and implementation of the project and contributed to the writing and overall revision of this manuscript. All authors read and approved the final manuscript.

## Pre-publication history

The pre-publication history for this paper can be accessed here:

http://www.biomedcentral.com/1471-2458/12/973/prepub
